# Immune-Mediated Thrombocytopenia Induced by Niraparib: A Clinically Relevant Case

**DOI:** 10.7759/cureus.97299

**Published:** 2025-11-19

**Authors:** Fernando Roxo, Delfim Duarte, Miguel H Abreu

**Affiliations:** 1 Department of Medical Oncology, Instituto Português de Oncologia do Porto Francisco Gentil, Porto, PRT; 2 Department of Hematology and Bone Marrow Transplantation, Instituto Português de Oncologia do Porto Francisco Gentil, Porto, PRT; 3 Hematopoiesis and Microenvironments Group, i3S-Instituto de Investigação e Inovação em Saúde, Universidade do Porto, Porto, PRT; 4 Department of Biomedicine, Faculdade de Medicina da Universidade do Porto, Porto, PRT

**Keywords:** adverse drug reaction, corticosteroid therapy, drug-induced thrombocytopenia, high-grade serous ovarian carcinoma, immune-mediated thrombocytopenia, myelotoxicity, niraparib, parp inhibitor

## Abstract

Immune-mediated thrombocytopenia secondary to niraparib is very rare; pharmacovigilance signals are scarce, and the true incidence is unknown. We report a 64-year-old woman, negative for BRCA pathogenic variants, with stage IIIC high-grade serous carcinoma of the ovary, fallopian tube and peritoneum, who started maintenance niraparib 300 mg/day after neoadjuvant chemotherapy and cytoreduction surgery. Platelets fell to grade 1 in week 1 and grade 2 in week 2, leading to interruption. One week later, she presented with grade 4 thrombocytopenia (2×10^3/µL) and petechiae. There was no anaemia or other cytopenias; smear showed no schistocytes; coagulation, direct antiglobulin test and viral serologies were negative. Dexamethasone 40 mg/day for four days restored counts. Re-challenge at 200 mg/day reproduced grade 2 thrombocytopenia by week 2 and again resolved with steroids; niraparib was permanently discontinued. One year post-surgery, she remains without recurrent thrombocytopenia or disease progression. This pattern supports an immune mechanism and underscores the need for steroid therapy and withdrawal.

## Introduction

Poly(ADP-ribose) polymerase (PARP) inhibitors are widely used as maintenance therapy in patients with high-grade serous ovarian carcinoma after response to platinum-based chemotherapy. Niraparib improved progression-free survival in first-line randomised trials (e.g., PRIMA) and is used irrespective of BRCA status [1]. Haematologic toxicity, particularly thrombocytopenia, is a recognised class effect and a frequent cause of dose modification [2,3]. Drug-induced immune thrombocytopenia (DITP) refers to an abrupt, immune-mediated fall in platelets triggered by drug-dependent antibodies; counts typically recover after drug withdrawal and/or immunomodulatory therapy [4,5]. While many events reflect dose-dependent myelosuppression, immune-mediated mechanisms (DITP) have been increasingly reported and warrant different management [4,5]. Distinguishing immune-mediated thrombocytopenia from marrow toxicity is critical because immune cases often respond promptly to immunomodulation and typically require drug discontinuation if reproducible on re-exposure.

## Case presentation

**Figure 1:**
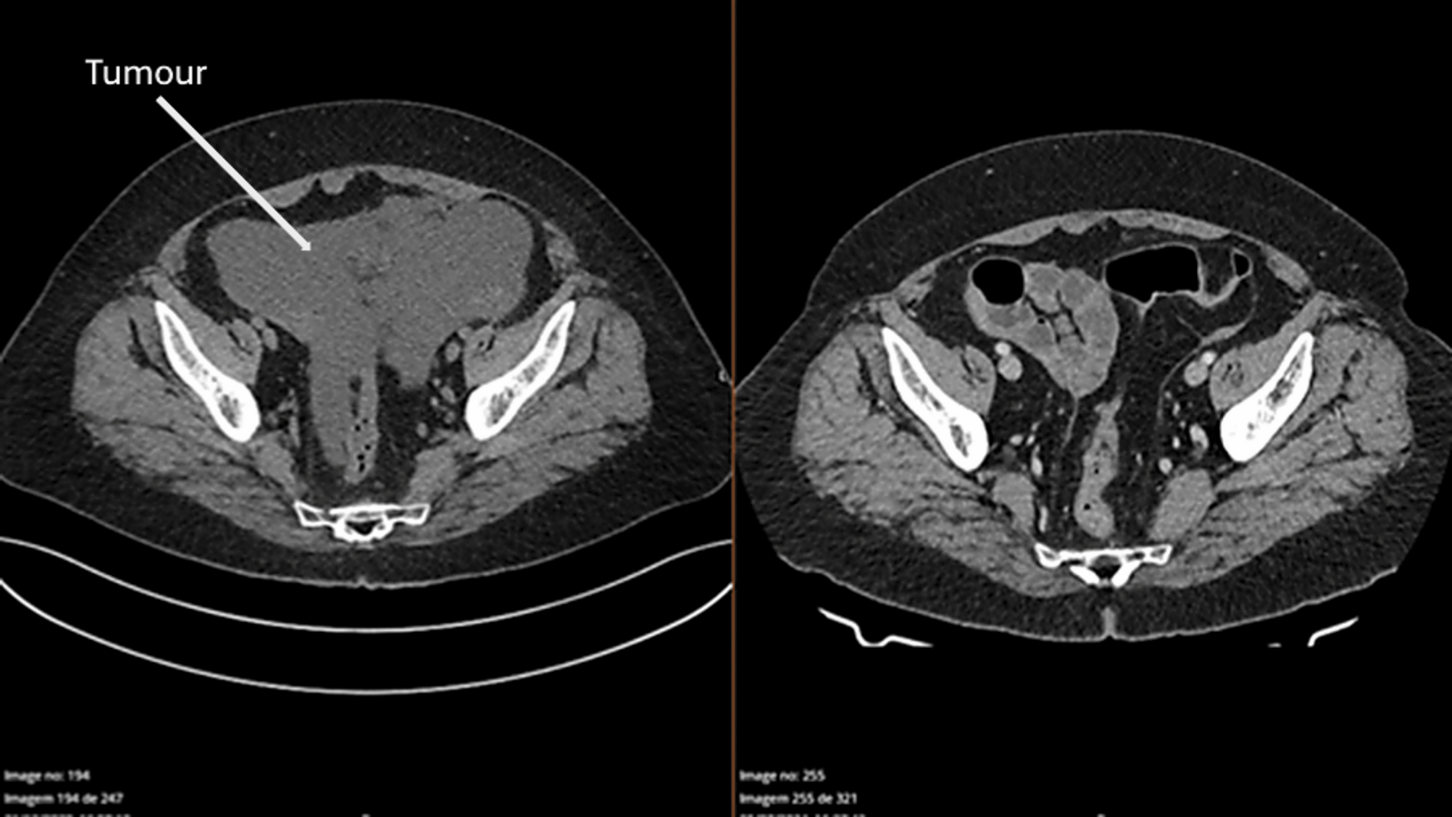
CT scan of the disease before and after chemotherapy Left: before chemotherapy; right: after chemotherapy

We describe the case of a 64-year-old woman, BRCA wild-type, with a past medical history of ulcerative colitis and hypothyroidism; her chronic medication included levothyroxine, mesalamine, bisoprolol, telmisartan, levetiracetam, aspirin, pitavastatin, and gabapentin. She was diagnosed with high-grade serous carcinoma of the ovary, fallopian tube, and peritoneum. On the initial staging magnetic resonance imaging, the patient presented large‐volume ascites, an omental cake, peritoneal metastatic implants and bilateral ovarian neoplasms, and was staged as IIIc FIGO (International Federation of Gynecology and Obstetrics) [6]. She started neoadjuvant chemotherapy, and interval cytoreduction surgery was only possible after six cycles (Figure 1).

Maintenance therapy with niraparib (300 mg/day) was then initiated; at this time, platelet count was 156 x10⁹/L. In the first week of treatment, grade 1 thrombocytopenia emerged and progressed to grade 2 in the second week, prompting suspension. One week later, she was admitted for grade 4 thrombocytopenia (platelet count 2000/L), with petechiae in the oral cavity and on the skin, but no active bleeding or fever (Table 1). Secondary causes were excluded through haematology work-up, specifically no anaemia or involvement of the other hematologic lineages, peripheral blood smear without schistocytes or other morphological abnormalities, coagulation studies without alterations, negative viral serologies and negative direct antiglobulin test (DAT). DAT was performed to rule out secondary immune cytopenias; the negative result supported an isolated immune-mediated thrombocytopenia rather than multisystem immune involvement. Corticosteroid therapy with dexamethasone 40 mg per day for four days was initiated, resulting in full platelet recovery.

**Table 1:** Admission and hospital day 1 haematological profile MCV: mean corpuscular volume

Parameter	16/10 admission	Hospital day 1
Haemoglobin (g/dL)	11.3	11.0
MCV (fL)	82.4	81.6
Leucocytes (/µL)	3,060	3,590
Neutrophils (/µL)	1,510	2,810
Platelets (/µL)	2,000	1,000

**Figure 2:**
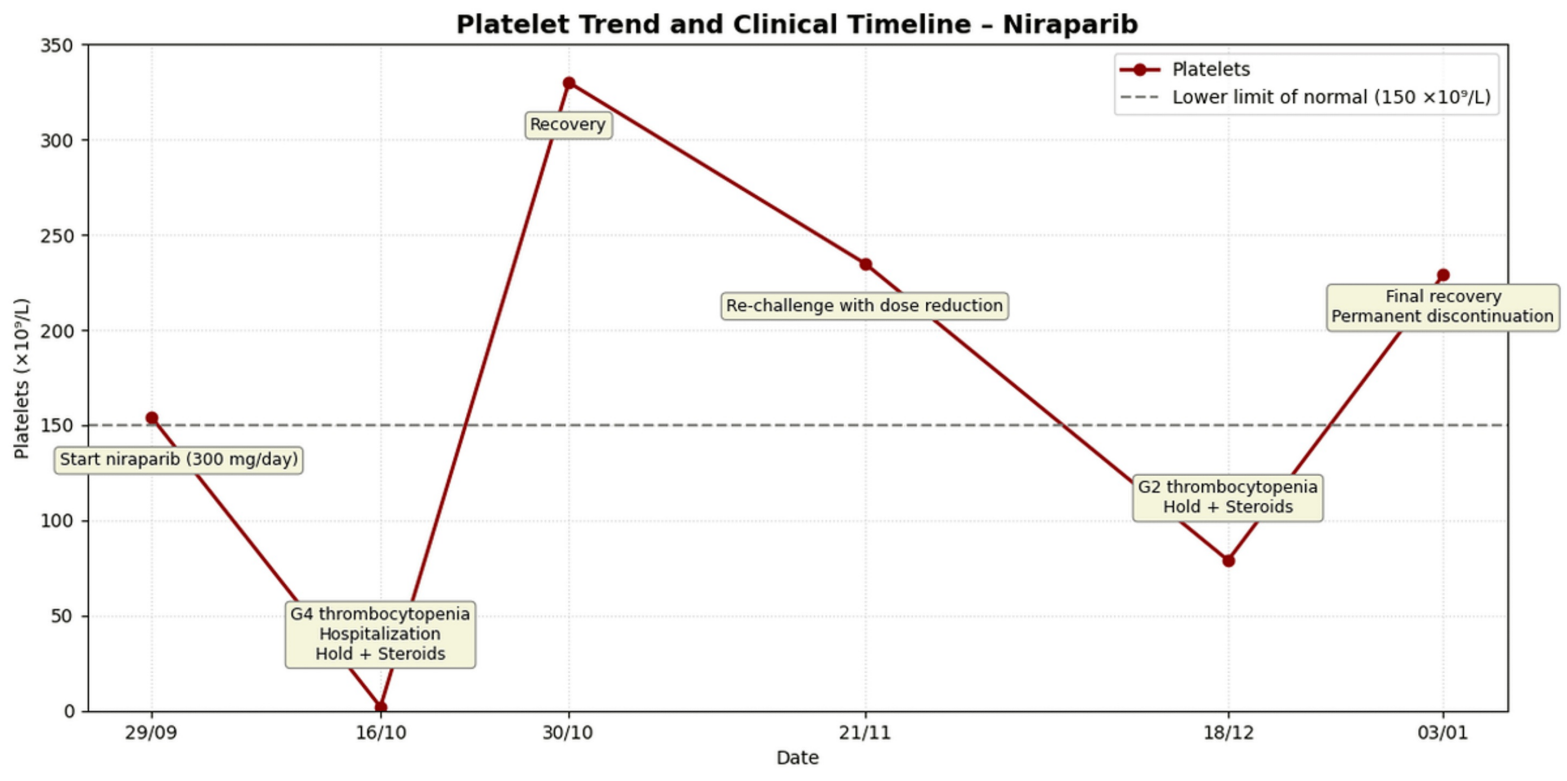
Platelet trend and clinical timeline

Re-challenge with a reduced dose of niraparib (200 mg/day) resulted in recurrent grade 2 thrombocytopenia by the second week, again responsive to a second cycle of dexamethasone. This recurrence, following a reproducible pattern, strongly supported an immune-mediated mechanism. Definitive suspension of niraparib was decided, and the patient remains in follow-up without recurrence of thrombocytopenia or signs of disease progression, one year after surgery and seven months after definitive suspension of niraparib (Figure 2).

## Discussion

The abrupt and reproducible pattern of platelet decline in our patient favours an immune-mediated mechanism over cumulative myelotoxicity. Thrombocytopenia is the main haematologic toxicity of niraparib; individualised starting doses and early monitoring help mitigate risk but do not fully prevent severe events [7]. Case reports and pharmacovigilance analyses have described severe thrombocytopenia temporally linked to PARP inhibitors, with variable responses to supportive measures; some series suggest immune mechanisms in a subset of cases [8,9]. Published cases of niraparib-associated severe thrombocytopenia are sparse but instructive. Li et al. reported a 59-year-old woman on niraparib maintenance who developed persistent, severe thrombocytopenia approximately six months into therapy; platelet counts fluctuated between 7-107 ×10⁹/L despite drug interruption, transfusions, interleukin-11 and thrombopoietin-receptor agonists, ultimately necessitating discontinuation [10]. In a three-patient series, acute refractory thrombocytopenia occurred shortly after niraparib exposure during paclitaxel/carboplatin; the authors considered an immune-mediated mechanism and noted benefit from early full-dose thrombopoietin-receptor agonists when standard measures failed [11]. These reports contextualise our observation as rare and support prompt evaluation for an immune phenotype when platelet counts drop precipitously under niraparib.

Although niraparib may be myelotoxic as part of its class effect [3], our case shows that it can also present with an immune-mediated thrombocytopenia. Myelotoxicity typically has a more chronic or subacute course, involves multiple lineages (via effects on haematopoietic stem/progenitor cells), produces less abrupt cytopenias, and is associated with a hypocellular and often dysplastic marrow. By contrast, immune thrombocytopenia is acute and severe, predominantly affects a single lineage, and often shows an increased number of megakaryocytes in the marrow. Although bone marrow evaluation was not performed, the peripheral blood profile and the rapid steroid responsiveness in our patient support an immune-mediated thrombocytopenia, with a possible contribution from myelosuppression.

Real-world and pharmacovigilance studies have highlighted the frequency and spectrum of haematologic adverse events associated with PARP inhibitors, including early severe thrombocytopenia in a subset of patients; analyses of the FDA Adverse Event Reporting System and other registries, as well as single-country cohorts (China, Japan), provide complementary evidence to clinical trials and underscore the need for active monitoring [8,9,12-14].

DITP is characterised clinically by abrupt platelet drops after drug exposure, exclusion of alternative causes, and recovery after drug withdrawal and/or immunotherapy [4,5]. Laboratory testing for drug-dependent platelet antibodies is available in reference centres but may not be accessible everywhere; clinical criteria often guide diagnosis and therapy [4]. Recommendations for immune thrombocytopenia include corticosteroids as first-line therapy and intravenous immunoglobulin (IVIG) for severe bleeding or when a rapid response is necessary; rituximab and thrombopoietin receptor agonists (e.g., eltrombopag, avatrombopag) are viable therapeutic options; evidence for their use in PARP inhibitor-related thrombocytopenia is currently limited to case reports and small case series [5,15]. In our case, dexamethasone (40 mg daily for four days) was selected rather than a prednisolone regimen because it carries comparable first-line evidence in adult immune thrombocytopenia and obviates the need for tapering [16]. IVIG was not administered, as it is typically reserved for adults with severe bleeding or when a rapid platelet rise is required. Our case strengthens the argument for immune pathogenesis because of the reproducible decline on re-exposure and steroid responsiveness.

## Conclusions

In patients receiving niraparib who experience an abrupt or severe platelet decline, clinicians should explicitly differentiate immune-mediated thrombocytopenia from cumulative marrow toxicity-considering timing, severity and single-lineage pattern, peripheral smear/IPF (immature platelet fraction) where available, and response to corticosteroids. Early corticosteroids (with IVIG when indicated) can reverse thrombocytopenia and prevent bleeding; re-exposure may reproduce the event even at reduced doses, in which case permanent discontinuation should be considered. Accordingly, PARP-inhibitor regimens should incorporate early and frequent platelet monitoring-particularly in the first four to eight weeks and after dose changes-and a low threshold for prompt haematology referral when counts fall or bleeding symptoms emerge.
